# NEWS

**DOI:** 10.7189/jogh.05.010203

**Published:** 2015-06

## DEMOGRAPHY

➢ Globally, the age of marriage is increasing, which translates into lower numbers of child brides. The proportion of young women married before the age of 18 fell from 33% in 1985 to 25% today, and the proportion married before the age of 15 fell from 12% to 8% over the same period. Higher age at marriage tends to reflect more freedom in choosing a spouse, and there is a 40% decline in arranged marriages across Africa and Asia. Increasing education, employment and urbanisation could be factors behind the decline in arranged marriages. Older marriages benefit development, as women are less likely to face violence, or HIV and HPV infections from their partner. Moreover, the mortality risk for young mothers (aged 15–19 years) is twice as high compared to older mothers, and the mortality risk for the infants of young mothers is 73% higher in comparison. (*Bloomberg*, 26 Jan 2015)

➢ China’s birthrate is one of the lowest amongst developing countries, and a recent change in the law allowed more couples to have a second child. In Shanghai, China’s biggest city, 90% of women of child–bearing age are eligible for a second child, but only 5% had applied to do so. An aging society could slow down China’s economic boom and strain its pension system, with too few workers supporting growing numbers of elderly people. The governor’s office of the populous Shandong province, called attention to another consequence of the country’s one–child policy – it has 20% more men than women, as many couples restricted to one child abort female fetuses until they have a male baby. Many Chinese couples are deterred from having more than one child as education and other costs soar, and birth rates have also fallen in neighbouring countries plus Hong Kong and Taiwan. (*The Guardian*, 29 Jan 2015)

➢ The world’s 300 largest metropolitan economies contribute nearly 50% of global economic output, with only 20% of global population. The Brookings Institution (USA) surveyed the fastest–growing metropolitan areas in 2015, measured by economic performance (including employment and GDP growth). The 10 fastest–growing areas were concentrated in China, Turkey and the Middle East. In China, the cities of Fuzhou, Xiamen, Hangzhou and Kunming were included, based on eg, strong manufacturing, growth in business services etc. In Turkey, Ankara, Bursa, Istanbul and Izmir featured thanks to diversified economies, strong industrial bases and geographical locations. Dubai featured due to its success in diversifying its economy away from commodities towards services. Macau topped the performance index thanks to its strong economic growth, and is now the world’s largest gaming centre. (*Brookings Institution*, 10 Feb 2015)

➢ In the UK, young adults (aged 20–24 years) have traditionally been wealthier than their elder peers (aged 65–69 years). However, this began to change in 2000–2001, when the living standards of young adults fell, and were overtaken by rising living standards of older adults. The recent recession accelerated this trend, and it is repeated across all socio–economic age groups (albeit more strongly for middle– and low–income families). Rising house prices, which have greatly benefited older people, is one of the main drivers behind this shift. There are few indications that the younger generation is closing the gap, with negative consequences for their financial prospects. Government policies have focused expenditure cuts on working–age rather than pensioner benefits – also sharpening the divide. Angus Hanton of the Intergenerational Foundation calls for government action to redistribute resources more fairly, saying “young people may want to tear up the social contract between generations.” (*Financial Times*, 23 Feb 2015)

➢ A UK parliamentary report by the Commons International Development Committee (IDC) warns of a “ticking time–bomb” of youth unemployment in developing countries, and globally 600 million young people are competing for 200 million jobs. It states that the problem must be taken as seriously as humanitarian disasters, and it risks widespread social and political unrest. It recommends the UK government’s Department for International Development (DfID) widen its focus beyond manufacturing, agriculture and food to include travel and tourism – which could potentially create 73 million jobs by 2025 – and considerable numbers could also be created in health and education. The IDC also says that DfID should have “specific interventions” to help marginalised groups benefit from economic growth, and help lift barriers to women and girls’ educational and employment opportunities. (*The Guardian*, 24 Mar 2015)

## ECONOMY

➢ Growing numbers of South Africans are using digital payment apps rather than cash, and digital payments are becoming more widely accepted by vendors ranging from street traders to large retailers. Digital retailers are driving down costs – many do not charge shoppers a transaction fee, and retailers pay a small fee which is comparable to, or cheaper than, a card transaction fee. More than 50% of South Africans own a mobile phone, so there is a vast potential market for digital payments. Digital payments could expand into other areas, such as church collections and car–parking fees. (*BBC*, 5 Dec 2014)

➢ In developing countries, the gender gap in access to formal financial services is great, with women 20% less likely than men to have a bank account. This gap, particularly marked amongst poorer people, impedes women from tapping into market opportunities and widens existing gender imbalances. If financial services are designed around women’s needs – convenience, reliability, security, privacy – improved access could lead to greater economic participation, empowerment, account ownership and asset accumulation. Mobile technology can facilitate this by providing services in remote areas, shifting financial decision–making towards women, and making it easier for women to receive remittances from husbands working elsewhere. However, many women do not possess mobile phones or know how to use them, so the products must be simple to use. Governments must also support suitable regulatory environments and remove discriminatory laws that impede women’s access to finance. (*OECD*, 6 Feb 2015)

➢ Between 2002 and 2013, 60 million people in Latin America moved out of poverty, and the poverty rate – the proportion of people living on less than US$ 4/day – fell. However, progress has stalled since 2013, and the region’s poverty rate remains stuck at 28%, whilst the proportion of people in extreme poverty (ie, living on less than US$ 2.50/day) rose slightly to 12%. With a per–person income of US$ 13 500, Latin America is an upper–middle income region, but faces extreme income inequality with large numbers of poor people. Reasons behind this faltering progress include slowing economic growth; less job creation; and poorer people benefiting disproportionately less from existing economic growth. Latin America now faces challenges of implementing more effective mechanisms for helping people out of poverty; and for ensuring that people can leave poverty permanently. Improved economic growth will help, but better policies and investments are needed alongside it. (*The Economist*, 19 Feb 2015)

➢ In a speech at the opening of the China Development Forum, Vice Premier Zhang Gaoli said that China will work to unleash new economic growth as China, to maintain a medium–high growth rate and transition to a medium–high development level. The government plans to expand economic reforms to spur growth arising from entrepreneurship, innovation and creativity. It will make China’s economy more open to the outside world in order to achieve prosperity and share development opportunities. China is moving away from the double–digit growth of the past decade towards slower but high quality growth – the “new normal”. (*Xinhau*, 22 Mar 2015)

➢ Demographic changes, the impact of the economic crisis, and the low–growth, low returns and low yields environment is presenting crucial and far–reaching challenges to pension systems of all sorts. The *OECD Pensions Outlook* discusses how these challenges are being addressed. Ageing, due to lower fertility and higher life expectancy, increases a population’s average age. This creates problems of adequacy, solvency and financial sustainability. The *Outlook* argues that contributing more, and for longer (eg, by increasing retirement ages) is the best approach. However, this could be unfair if gains in life expectancy are distributed unevenly, and linking the number of years’ contribution may be more equitable. Uncertainties over future improvements in mortality and life expectancy can be overcome by transferring the risk to annuity providers (eg, life insurers). Pension funds and annuity providers will need regularly updated mortality tables that incorporate future predictions, based on trends in the relevant population. A regulatory framework that ensures capital markets can mitigate longevity risk, eg, by index–based instruments to hedge risk will also help. (*OECD*, 9 May 2015)

## ENERGY

➢ 1.2 billion of the world’s poorest people lack mains electricity, and a further 2.5 billion people have inadequate power. Sub–Saharan Africa, with 910 million people, consumes less electricity than Alabama, USA – equivalent to one light bulb per person for 3 hours/d. Without electricity, people rely on paraffin – expensive and dangerous, with indoor fumes causing 600 000 preventable deaths in Africa alone. However, new technologies are providing potential solutions as Africa’s population is set to double by 2040. Solar power is now cheaper to generate, falling from US$ 4/watt in 2008 to US$ 1 in 2014. Light emitting diodes – highly efficient at converting electricity to light – have also fallen in price, and more solar–powered devices and systems are available. Quality, technical and capital constraints curtail wider up–take – although the International Energy Authority estimates that 500 million people without electricity will have at least 200 W/person by 2030 thanks to solar power. (*The Economist*, 15 Jan 2015)

➢ Nepal cleared China’s Three Gorges International Corp. to build a US$ 1.6 billion hydropower project – its single–biggest foreign investment. The dam will be built on the West Seti River in northern Nepal, and will generate 750 megawatts (MWs) of power upon completion in 2021–2022. Nepal, one of the world’s poorest countries, is opening up its vast hydropower potential to ease power shortages and strengthen its war–torn economy. This has led to China and India to invest in Nepal’s energy–building, and India has begun exporting energy. Nepal could potentially generate 42 000 MW of hydropower, but currently produces 800 MW – much less than the demand of 1 400 MW. In 2014, Nepal gave clearance to two major India hydropower projects worth a combined US$ 2.4 billion. China is investing in other infrastructure projects, including the road linking the capital Kathmandu with the Tibetan border. (*Reuters*, 13 Apr 2015)

➢ Since 2013, the world has added more renewable energy capacity than coal, natural gas and oil combined. According to an analysis presented at the Bloomberg New Energy Finance (BNEF) Summit, this trend will accelerate, and by 2030 more than four times as much renewable capacity will be added – despite recent falls in oil and gas prices. Falling wind and solar prices mean that they are now cheaper, or equivalent to, grid electricity in much of the world, and solar power could be the world’s biggest energy source by 2050. However, BNEF warns that projected investment levels in renewable energy fall short of averting a global temperature increase of 2°C, and hence the most severe consequences of climate change. (*bloomberg.com*, 14 Apr 2015)

➢ The European Commission (EC) has sent a Statement of Objections to Gazprom, the Russian state–owned gas company, over its alleged abuse of market power in Central and Eastern Europe gas markets. Gazprom denies these claims. This is part of a wider anti–trust case that was initially opened in August 2012. The EC believes that Gazprom has broken EC anti–trust rules by “pursuing an overall strategy to partition Central and Eastern European gas markets, with the aim of maintaining an unfair pricing policy in several of these member states’ by: the possible hindering of cross–border gas sales; alleged unfair pricing policy; and concerns over gas transport infrastructure. However, the EC could be applying its competition policy in under–developed markets, making it difficult to prove market abuses – and unfair pricing practices. Likewise, infrastructure is a government responsibility, not Gazprom’s. If the EC complaint is upheld, Gazprom can be fined up to 10% of its annual revenues. (*Brookings Institution*, 23 Apr 2015)

➢ According to a report by the International Monetary Fund, energy subsidies – at US$ 4.2 trillion in 2011 – are much higher than previously estimated, and are equivalent to 5.8% of global GDP. These subsidies are set to grow to US$ 5.4 trillion (6.5% of global GDP) by 2015, despite falling energy prices. This is more than is spent on social welfare (estimated at US$ 1.4 trillion, or 2% on global GDP in 2013). High levels of subsidies on environmentally–damaging coal and petroleum energy sources account for a large share in the overall cost. Reforming these subsidies would not only be environmentally beneficial, but would enable expenditure to be re–directed towards social welfare, eg, reducing labour taxes or increase expenditure on education and health. (*International Monetary Fund*, 11 May 2015)

## ENVIRONMENT

➢ According to the UN World Meteorological Organisation (WMO), 14 out of the 15 hottest years on record have occurred since 2000, with 2014 being the hottest year on record since 1850. The average global air temperature over land and sea in 2014 was 0.57°C above the average of 14°C for the 1961–1990 reference period. The record temperature was above temperatures in 2005 and 2010, albeit within a margin of uncertainty. The WMO secretary–general, Michel Jarraud, expects global warming to continue due to rising levels of greenhouse gases and the increasing heat content of the world’s oceans. The WMO also noted that 2014 temperatures occurred despite a fully–formed El Nińo event, which drives up temperatures. The confirmation of 2014’s extreme heat comes ahead of the next round of UN climate negotiations in Geneva, which are intended to pave the way towards a global agreement to tackle climate change. (*The Guardian*, 2 Feb 2015)

➢ Taiwan experienced its lowest winter and autumn rainfalls since 1947, leading to its worst drought for 70 years. As a result, Taiwan introduced water rationing in some cities to deal with urgent water shortages. Homes, schools and business are reliant on water stored in tanks, and water–saving measures (eg, recycling water, closing swimming pools and gyms), are being adopted. Despite some light rainfall, the government warned that the dry spell is set to continue, and that monsoon rains may not happen this season. (*The Guardian*, 8 Apr 2015)

➢ Under international law, people leaving their countries due to natural disasters or environmental degradation are classed as normal migrants, rather than refugees fleeing persecution. This may become more pressing, as one of the effects of climate change will be more frequent, intense and unpredictable natural disasters. The Nansen Initiative was sent up to explore this gap in international law, and highlights how little is known about future migratory patterns. There are widely varying estimates of how many people will move within their countries, or cross national borders as a result of natural disasters and climate change. Uncovering the risk of displacement from these events is complex, as environmental and economic migration can intersect. Elizabeth Ferris, Co–Director of the Brookings/LSE Project on Internal Displacement, calls for policies and mechanisms that will help those who cannot survive in their own country, and will seek to enter another country. (*Brookings Institute*, 22 Apr 2015)

➢ Ahead of a UN summit in Paris on limiting climate change, a new study found that global warming is responsible for 75% of moderate hot extremes and 18% of downpours. 2014 was the warmest year on record since the 19th century, and heavy flooding hit several countries including Serbia, Bangladesh and Morocco. Global average temperatures have risen 0.85°C above pre–industrial levels, and further warming would increase the risk of extremes. The authors warn that a temperature increase of 2°C from pre–industrial times would raise the share of heat extremes attributable to warming to 96%, and the share of extreme rainfall to 40%. (*Yahoo*, 27 Apr 2015)

➢ A study published in the journal *Science* outlines threats to soil productivity – and hence food production – arising from soil erosion, nutrient exhaustion, urbanisation and climate change. The soil layer is only 1m thick, and has a critical life–support role. 40% of the planet’s terrestrial surface is used for agriculture, whilst other large – and growing – areas are urbanised. As the remaining land is less suitable for cultivation, the study’s authors recommend more effective use of current land resources in order to support the world’s growing population. Soil is eroding more quickly than production, resulting in the loss of key nutrients and the unsustainable use of chemical fertilisers. “Unless we devise better ways to protect and recycle our soil nutrients and make sure that they are used by crops efficiently rather than being washed away, we are certainly headed for nutrient shortages,” says Donald Sparks, one of the report’s authors. He also noted that disruptions of food production could become a source of conflict. (*Science Daily*, 7 May 2015)

## FOOD, WATER AND SANITATION

➢ Senegal’s capital city, Dakar, will be the test site for the new Omniprocessor technology. The Omniprocessor is a compact waste treatment plant that can process sewage for a community of 100 000 people. Conventional sewage plants use large amount of electricity to process waste, but the Omniprocessor combines incineration, steam power and filtration techniques to ensure no energy is wasted in the process. It also generates 11 000 L of drinking water in the process, and derives enough energy from the faecal matter it incinerates to run the unit, with an additional 150 kw/d to export to the national grid. Its ash can be commercially valuable as a fertiliser. The system’s ability to generate revenue will support its running costs – crucial when sewage systems are abandoned when countries cannot afford maintenance. It is a potential weapon in tackling the problem of 2.5 billion people without sanitation – poor sanitation is one of the main reasons behind 1.5 million children dying each year from diarrhoea. (*The Guardian*, 20 Jan 2015)

➢ A report from the Waste and Resources Action Program (WRAP) showed that 60 million tonnes of food is wasted each year in the USA, with an estimated value of US$ 162 billion – and 32 million tonnes end up in landfills, at a cost of US$ 1.5 billion to local governments. This problem is not confined to the USA – it estimates that globally, one–third of food is never consumed, costing up to US$ 400 billion annually. Reducing food waste to 20% could save US$ 120–130 billion a year by 2030, and the UN Food and Agriculture Organization states that food waste by retailers and consumers in developed countries is sufficient to feed the world’s 870 million hungry people. Food waste is expected to increase with the growing global population, and this has environmental costs (eg, water and energy usage). Food landfills also emit methane, a greenhouse gas, so reducing food waste could also reduce global warming. WRAP applauds efforts to reduce food wastage, but calls for more preventative measures. (*New York Times*, 25 Feb 2015)

➢ A new report from the WHO and WaterAid shows that globally, 500 000 babies die in their first month of life from preventable deaths in unclean hospitals and clinics. Sepsis is a major killer in the first month, and can be prevented by hand–washing, washing the baby in clean water, and making sure the instrument used to cut the umbilical cord is clean. The report showed that more than one–third of such centres in developing countries lack hand–washing or toilet facilities, and nearly 50% in Africa lack access to clean water. Water supplies, where they exist, are often unreliable and inaccessible. Lack of water and sanitation contributed to the spread of Ebola; with pressures on inadequate health systems from malaria and diarrhoea – both related to, and spread by lack of sanitation – made it more difficult to tackle Ebola effectively. (*Newsweek*, 17 Mar 2015)

➢ A UN report published ahead of World Water Day on 22 March states that the world will face a 40% shortfall in water supply by 2030 unless there are major improvements in water management. Overall, demand for water will increase by 55% by 2050. The output from agricultural and manufacturing sectors – both intensive water consumers – will rise due to increasing population. It expressed concern over decreasing groundwater supplies, and the contamination of groundwater by seawater due to rising sea levels. It noted that 748 million people lack access to improved drinking water, with poor people, the disadvantaged and women more likely to be affected. A US$ 53 billion investment – less than 0.1% of global GDP – in water and sanitation could give universal access, and reap substantial economic benefits. It also called for the sustainable development goals to include governance, water quality, wastewater management and the prevention of natural disasters. (*LiveMint*, 20 Mar 2015)

➢ At a G20 meeting on food security and nutrition, agriculture ministers stated that food waste is a huge economic problem. Each year, an estimated 1.3 billion tonnes – 30% of global food production – is lost or wasted, which could easily feed the world’s 800 million hungry people. The ministers said countries need better estimates of food waste and the economic impact of food loss to help fight the problem. (*The Guardian*, 8 May 2015)

## PEACE AND HUMAN RIGHTS

➢ 2014 saw the rise of the Islamic State in Iraq and Syria, Russian forces assaulting Ukrainian borders, civil war in South Sudan and Boko Haram dangerously undermining Nigeria’s sovereignty. However, there are signs of hope with Senegal and Nigeria’s rapid Ebola response, Lebanon absorbing thousands of Syrian refugees and Afghanistan’s marginally–brighter prospects. Out of several countries showing democracy, resilience and potential, *The Economist* identified two main contenders for its Country of the Year – Indonesia and Tunisia. Indonesia’s recently–elected reforming president, Joko Widodo, is steering his country towards prosperity. The winner, Tunisia, is a shining exception to the squandered Arab Spring, when it adopted an enlightened constitution and held both parliamentary and presidential polls. *The Economist* lauded Tunisia for its pragmatism and moderation that engender hope in a troubled world. (*The Economist*, 20 Dec 2014)

➢ US Senators introduced the End Modern Slavery Initiative Act of 2015, and called for a US$ 1.5 billion fund to combat global slavery through more rigorous law enforcement and prosecution of human traffickers. Slavery and human trafficking and estimated to generate US$ 150 billion profits from the exploitation of 30 million people. The bill aims to cut slavery by 50% over seven years wherever it is most prevalent. The International Justice Mission has documented large reductions in trafficking with modest investments in law enforcement. The Act would authorise the establishment of a non–profit foundation that assists victims, creates preventions programmes and strengthens law enforcement. The US would contribute US$ 251 million in seed money, with an additional US$ 500 million from other governments and US$ 750 million from the private sector. (*Thomson Reuters Foundation*, 25 Feb 2015)

➢ The UK–based charity Death Penalty Project (DPP) provides free legal representation by volunteer barristers to people condemned to death around the world. Using “the law to change the law”, the DPP campaigns against the death penalty by meticulous objections and reasonable restrictions, and always working within each country’s rules. It has saved hundreds of people from executions, making future executions less likely in the process. The DPP has succeeded in having the death penalty declared unconstitutional in Uganda and three Caribbean countries, and mandatory death sentences declared unconstitutional in Kenya and Malawi. The DPP campaigns to have proper policing recognised as a more effective deterrent than capital punishment, and the stated aim of its founders, Parvais Jabbar and Saul Lahrfreund, is “to put themselves out of jobs”. (*Intelligent Life*, 11 Mar 2015)

➢ More than two–thirds of Syrian school–age refugees in Lebanon are not attending school, usually because their families cannot afford the fees or because they need to work to support their families. Sometimes, no schools are available, or children lack transport to attend school. It is very difficult for children who miss years of schooling to catch up on their education, and organisations such as World Vision are working to enable more refugee children to attend school. Their programmes include accelerated learning for “out–of–school” children, remedial classes, and informal education. They are also supporting teachers and providing more teaching infrastructure. However, these efforts fall short of what is needed, and a whole generation of children is growing up displaced and uneducated – with real, long–term impacts on them and their country – and providing access to education is something the world should not ignore. (*The Guardian*, 15 Mar 2015)

➢ According to the UN, the number of refugees seeking asylum in developed countries is at its highest level for 22 years, rising by nearly 50% in 2014, with an estimated 866 000 asylum seekers lodging claims. The increase is caused by the conflicts in Iraq and Syria, which have created the “worst humanitarian crisis of our era,” according to UNHCR spokeswoman Melissa Fleming. These figures do not include Syrian refugees who have been taken in by countries such as Lebanon and Jordan. She urged European countries to open their doors, and respond as generously to the current situation as they did in the Balkan wars in the 1990s. (*BBC*, 28 Mar 2015)

## SCIENCE AND TECHNOLOGY

➢ Scientists at the World Economic Forum in Davos called for changes to the development and licensing of new cancer drugs, to enable countries to cope with cancer’s increasing burden. Pharmaceutical companies should cut prices in return for a system where promising drugs can be licensed earlier without full–blown and expensive clinical trials, and prices increased if there is sufficient patient benefit. Other countries are looking at the US Food and Drug Administration’s breakthrough therapy designation, which accelerates approval of drugs for life–threatening conditions when benefits outweigh risks. Globally, cancer costs US$ 2 trillion a year in lost output and treatment costs – equivalent to 1.5% of global GDP – and pharmaceutical spending doubled to US$ 91 billion in 2013. “The current system for drug discovery and development is failing to deliver enough genuinely innovative advances in treatment, or to produce new drugs at a cost that is affordable for taxpayers,” says Paul Workman of the UK’s Institute of Cancer Research. (*Financial Times*, 25 Jan 2015)

➢ Pharmaceutical companies are accused of restricting access to drug treatments for Hepatitis C in developing countries. Gilead allows cheap copies of its drug sofosbuvir to be made by generic companies and sold in developing countries. However, Médecins Sans Frontiéres (MSF) argue that Gilead is imposing unacceptable conditions in its efforts to ensure that cheap generic copies do not reach developed countries. According to MSF, these conditions – which include requiring patients to prove identification, citizenship and residency – penalise refugees and marginalised groups. Anand Grover, a former UN special rapporteur on the right to health, agrees that these measures are contradictory in improving access to crucial drugs, and arguably, a violation of the right to health. Gilead responded by stating that these measures are designed to prevent the drugs from being diverted from their intended recipients. (*The Guardian*, 20 Mar 2015)

➢ According to a study published in the journal *Protein and Cell*, biologists in China have carried out the first experiment to alter the DNA of human embryos, whereby enzymes are introduced that bind to a mutated gene, eg, one associated with disease – and then repair or replace it. There are considerable ethical concerns over such work, as altering the DNA of human sperm, eggs or embryos could produce unknown effects on future generations, as the changes will be passed onto their offspring. This “germline editing” is a separate technology from “germline engineering” which alters the DNA of non–reproductive cells to repair diseased genes. Edward Lanphier (Chief Executive of Sangamo BioSciences Inc, and part of a group of scientists which has called for a global moratorium of such experiments) fears that this call is being ignored, and that it will be “the first of what may be many papers” on human germline engineering. (*Reuters*, 23 Apr 2015)

➢ Globally, 44 million people suffer from dementia, and current models of innovation have not delivered effective treatment for them. However, the revolution in digital health data (driven by technological developments, eg, broadband access, cloud computing and smart phones) could help address this. Dementia is clinically and biologically complex, so the studies required to underpin drug development need massive and diverse data collection, storage and processing. This data are being generated, and harnessing it would have wide–reaching benefits, but researchers’ willingness to share data are often constrained by other factors. First, patients’ informed consent tends to be limited to the primary study focus, and therefore often excluded for further studies. Step–by–step or dynamic consent models could address this. Second, scientists face disincentives in disclosing data, especially at pre–publication, so action is needed to boost data access and openness. Third, large–scale investment is needed in the necessary big–data infrastructure. Without better data sharing and knowledge co–ordination, there will be limited progress in understanding neurodegenerative diseases and treatments. (*OECD*, 1 May 2015)

➢ According to research published in *Science*, the measles vaccination prevents measles–induced immune system damage which makes children more vulnerable to other infectious diseases. The measles pathogen destroys immune system cells which “remember” how to attack other, previously–encountered, pathogens for 28 months after infection. During this time, children are more likely to die from other infections (eg, sepsis, pneumonia, and bronchitis). Prior to the introduction of measles vaccination in developed countries, measles may have been involved in 50% of childhood deaths from infectious diseases. This has important implications for maintaining high levels of measles vaccination, as the benefits are wider– reaching than protection against measles. (*Scientific American*, 7 May 2015)

